# Morphological, physical, and chemical characterization of coconut residues in Ecuador

**DOI:** 10.1016/j.heliyon.2023.e19267

**Published:** 2023-08-18

**Authors:** Gina San Andrés, Sara Aguilar-Sierra, Bernardo Graziella

**Affiliations:** a Universidad San Gregorio de Portoviejo, Ecuador; b Universidad Católica Luis Amigó, Colombia; c Università degli Studi della Basilicata, Italy

**Keywords:** Coconut fiber, Coconut endocarp, Solid vegetable waste, Circular production

## Abstract

In agricultural countries, Agro-industrial residues are better treated with a circular production logic. This article analyzes the morphological, physical, mechanical, thermal, and chemical characteristics of the coconut fiber and endocarp in Portoviejo and Rocafuerte Cities, Ecuador, to establish a baseline of knowledge that can help to incorporate coconut residues into a matrix of a new material. Interviews were conducted to coconut producers in Portoviejo and Rocafuerte and a random sample of the residues was collected from these locations. Analyses were performed using the SEM, TGA and FRX tests. Concerning the fiber, it was observed a tubular morphology with concentric microfibrils, specific weight 0.69 g/cm^3^, tensile strength 228 MPa, modulus of elasticity 3.03 GPa, thermal behavior with important mass losses at 330 °C, in the calcinations to obtain oxides as SiO_2_, SO_3_, Al_2_O_3_, Fe_2_O_3_ at 600 °C and 650 °C. The endocarp has a morphology of superimposed and consolidated smooth layers, specific weight 1.29 g/cm^3^, loss of mass at 339 °C and in calcinations at 800 °C. This study can help to address solid vegetable waste managing challenges in coconut producing cities.

## 1 Introduction

**Fig. 1:**
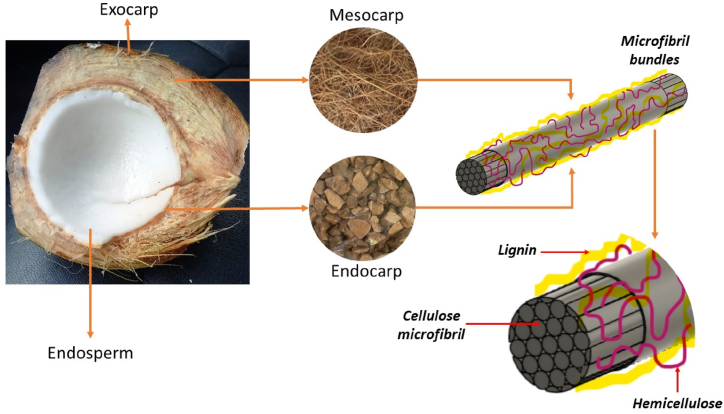
Coconut composition.

Cocos nucifera L. is the scientific name for the monocotyledonous class palm tree where coconut fruit grows [1,2]. This species is found on islands in tropical coastal areas around the world [3], with an average temperature range between 28 and 35 °C [2]. The fruit of the coconut palm tree is a monospermic drupe, composed of exocarp (coconut skin), 35% mesocarp (fiber), 12% endocarp (copra), 28% endosperm (meat) and 25% water [2,4]. Cellulose, hemicellulose, and lignin are the main components in the mesocarp and endocarp [[5], [6], [7], [8]]. The fiber comprises cellulose microfibrils embedded in a matrix of lignin and hemicellulose [9,10]. Fig. 1 illustrates the composition of the coconut.

Coconut residues have proven useful in the creation of various materials, with applications ranging from gardening products and handicrafts to briquettes [8]. With excellent hygrothermal performance, coconut fibers have found usage in green roof constructions [11]. These fibers can also serve as thermal insulators [12] and have been used as activated carbon within ceramic membrane support matrices [13].

The diverse properties of coconut fiber make it a key constituent in a range of material designs, including those intended for energy absorption and composite materials [4,14]. For instance, concrete has been reinforced with coconut fiber to enhance its strength and durability [15,16]. Furthermore, coconut fiber has been incorporated into reinforced epoxy polymer composites, contributing to their robustness [17,18].

A notable application of a composite material derived from coconut fiber and copper slag has been observed in the construction of ceiling boards [19]. Additionally, the combination of sludge and coconut fiber biochar has demonstrated stability against heavy metals and an ability to adsorb the antibiotic Ciprofloxacin from water [20]. Aerogel-based biosorbents from coconut fibers are all promising candidates for the treatment of contaminated wastewater [21]. These proposed applications showcase the potential for efficiently incorporating coconut residues into new composite material matrices.

In Ecuador, coconut production helps to sustain the economy of the northern coastal provinces of Manabí and Esmeraldas [22,23]. However, the lack of official data for this activity stands out. In Ecuador, the last agricultural census occurred in 2000. The figures that the Ministry of Agriculture and Livestock is relying on are based on an estimation by the National Institute of Census, which estimates a coconut palm planted area of 9134 ha. Although referential, this is the only official data that governmental institutions have at hand. An underlying problem that this study aims to address is the volume of waste generated by this industry and unused, despite its potential [23,24]. This study addresses an inherent issue, the large volume of waste generated by this industry, which remains unused despite its potential.

Previous investigations have revealed that coconut residues in Manabí are often incinerated in the open [25], and in the cities of Portoviejo and Manta, these wastes are discarded into sanitary landfill pits without prior waste classification [24].

This research focuses on the fiber and endocarp of coconut waste generated by the bottled juice industry in the cantons Portoviejo and Rocafuerte, in Manabí Province. These two cantons add up an approximate weekly production of 95 t. The most consumed type of coconut is the Manila, followed by the Hybrid and, to a lesser extent, the Manilon and Criollo. Specifically, the present investigation studies the morphological, physical, chemical, mechanical and thermal properties of the coconut endocarp and mesocarp and explores possibilities for its reuse.

## 2 Materials and methods

The study started by conducting thirty interviews with farmers and entrepreneurs from the areas of San José de Peñas, Sosote, El Resbalón, Danzarín in Rocafuerte Canton, and Río Chico and Abdón Calderon in Portoviejo Canton. The aim was to identify the species of coconut palms planted in these regions. Interviewees were chosen based on the information provided by the municipal governments of both cantons, with priority given to those who owned the largest coconut plantations. The plantations under consideration for this study spanned a total area of 315 ha.

**Fig. 2:**
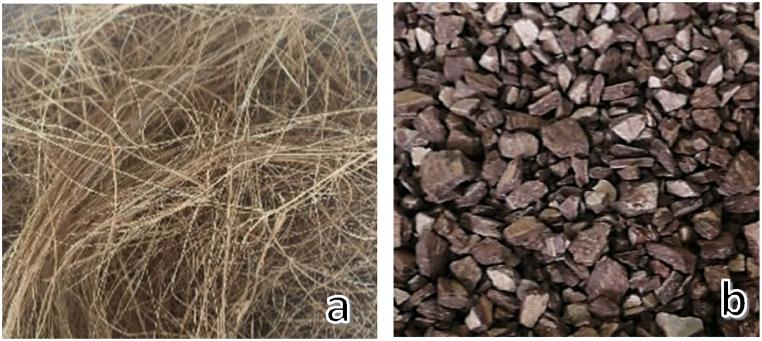
(a) Fiber obtained from the shredding machine. (b) Endocarp crushed and sieved.

Coconut residues selected for examination were randomly picked from the Abdón Calderon parish in Portoviejo Canton. The coconut fiber was extracted using a shredding machine. To further refine the material, the endocarp was crushed with a 4.5 kg proctor hammer and then sieved through a Los Angeles machine for granulometry, using sieves ranging from #4 to 30 (as shown in Fig. 2 a, b).

The morphological characterization of the samples was performed using a JEOL JSM 6490 LV scanning electron microscope. The samples were fixed on graphite tape and then coated with gold using a Denton Vacuum Desk IV. The morphology and topography of the samples were evaluated using a secondary electron detector. Elemental analysis was conducted using an EDX-X-ray microprobe (INCA PentaFETx3 Oxford Instruments reference).

Physical properties, including length, diameters, and specific weight, were measured using a metal ruler and a Leica DM 750 type optical microscope. The specific weight was calculated using equation (1). γ = Pd/V [5]. A total of fifty fiber samples were characterized, with three diameter measurement points taken for each sample.

Mechanical properties, such as tensile strength and modulus of elasticity, were assessed using a Shimadzu UH-F500Knx universal machine, with a precision of 0.00001. The tensile strength was calculated using equation (1); the deformation unit, using equation (2); and the modulus of elasticity of the fibers, using equation (3), in compliance with the ASTM D3822 tensile test standard.

(1) $$\sigma =\frac{F}{A_{0}}$$

(2) $$\varepsilon =\frac{\Delta l}{l_{o}}$$

(3) $$E=\frac{\sigma }{\varepsilon }$$

σ = Tensile strength

ε = Deformation unit

E = Modulus of elasticity

F = Strength

Δl = Variación de longitud

σ = Tensile strength

$A_{0}$ = Área inicial

$l_{o}$ = Longitud inicial

ε = Deformation unit

Eq. (1) Tensile strength; Eq. (2) Deformation unit; Eq. (3) Modulus elasticity.

For thermal characterization, Thermogravimetric Analysis (TGA; Q500, TA Instrument, Inc.) was performed between 23 and 900 °C at a rate of 10 °C/min under a nitrogen atmosphere.

Chemical characterization was performed using a combination of Scanning Electron Microscopy (SEM) and X-ray Fluorescence (XRF) on a Thermi ARL Optim'XWDXRF device. The analysis was semiquantitative, using Uniquant software under conditions of 41% humidity and a temperature of 24.7 °C. The ash samples for the XRF were labeled Fa, Fb, Fc, Fd, and Fe for the coconut fiber calcined at 500 °C, 550 °C, 600 °C, 650 °C respectively, while the endocarp samples were labeled E1, E2, E3 and calcined at 700 °C, 750 °C and 800 °C respectively. A sample of strong Portland Holcim cement, Gu type, was also analyzed as a reference.

## 3 Results and analysis

### 3.1 Types of coconut and its residues

**Fig. 3:**
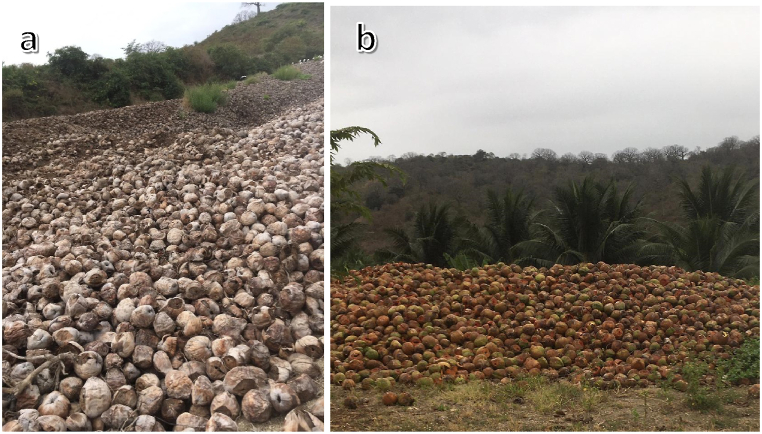
Images of coconut residues (a) Abdón Calderón parish in Portoviejo, June 2021 (b) Sosote de Rocafuerte site, July 2021.

This research was motivated by the findings from the interviews and on-site visits to coconut plantations and collection centers. A startling observation from these engagements was the minimal use of the coconut residues, as depicted in Fig. 3 a,b. Preliminary data from the Ministry of Agriculture and Livestock indicated that in 2019, Ecuador's annual coconut production was 6.2 tons, with 1.8 tons originating from Manabí. However, this official statistic seems significantly underestimated, considering the information from the conducted interviews, where one company alone reported producing 90 tons per week.

The interviewees consistently identified the most harvested types of coconuts: Manila (36.5%), Hybrid (30.2%), Manilon (28.0%), and Creole (5.3%). The preference for Manila coconuts was attributed to the sweet taste of its water. Meanwhile, Hybrid and Manilón varieties were favored for their larger size. The Creole variety's cultivation, however, was reported to be suffering due to pest issues.

### 3.2 Morphological characterization

To characterize the morphology of the coconut fibers and endocarp, the images obtained in the SEM scanning electron microscope were analyzed.

**Fig. 4:**
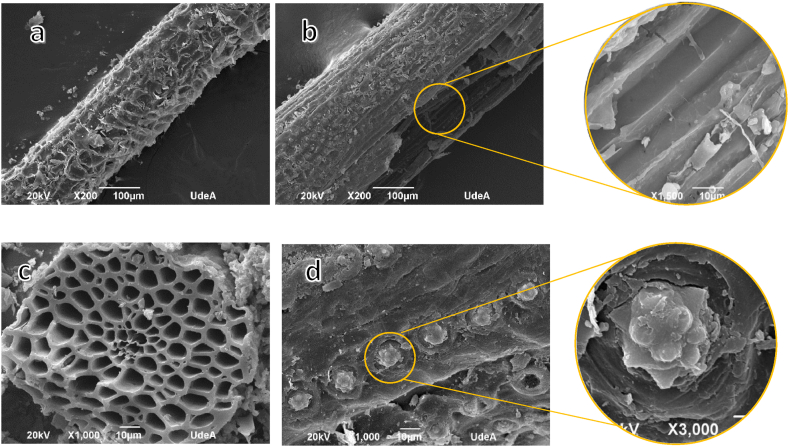
SEM images of coconut fiber. Squamous surface (a), tubular morphology (b), concentric microfibrils (c), flake-like silicon content (d).

In Fig. 4a, the Fc sample is observed showing the roughness with reliefs formed by flake-like layers between 30 and 50 μm long and 15–40 μm wide. In Fig. 4b, it is seen that its internal morphology is tubular, longitudinally juxtaposed with thicknesses between 8 and 22 μm, with thickness differences in the same microfibril. The cross section of a fiber Fig. 4c shows its tubular morphology with the formation of concentric microfibrils of various sizes, with cell widths between 1 and 11 μm. In Fig. 4d, on the surface of the fiber, sucker-type pits are observed that store the silicon particles with areas between 180 and 230 μm^2^, where the silicon particles have areas between 70 and 87 μm^2^ and the separation between the silicon flakes varies between 13.5 and 15.5 μm.

**Fig. 5:**
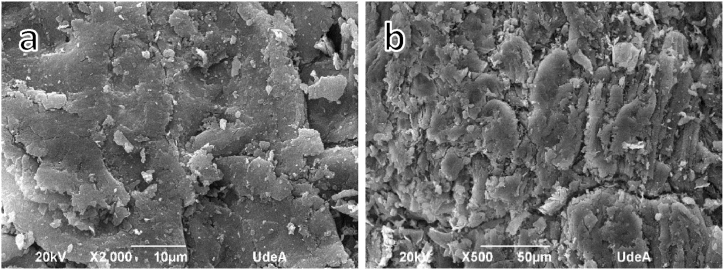
SEM images of the coconut endocarp overlapping flat layers (a,b).

Fig. 5 Indicates the morphology of the endocarp surface. Images 5a and 5b show that the endocarp surface is composed of superimposed layers that cause smooth unevenness, with approximate areas between 1068 and 1917 μm^2^, the middle layers approximately 170 μm^2^ and smaller layers around 20 μm^2^.

The consideration of attributes such as reliefs, shapes, and textures is crucial when incorporating these materials into a composite matrix. This is primarily because the adhesion between the reinforcing fiber and the matrix significantly impacts the performance of composite materials [26].

### 3.3 Physical characterization

The physical characteristics and properties of natural fibers depend on growth [27] and environmental conditions. For length measurement, 50 fiber samples were used, obtaining an average length of 196 ± 1 mm, the average diameter 0.31 ± 0.08 mm and the specific weight 0.69 g/cm^3^. Regarding the endocarp, 50 samples were taken, resulting in a thickness of 3.5 ± 1 mm, and specific weight 1.29 g/cm^3^. The results obtained are within the ranges of data from previous investigations [5,16,17,28,29,30].

### 3.4 Mechanical characterization

For mechanical characterization, we tested 20 fibers from 150 to 200 mm in the universal machine, at speed of 1 mm/min. The tensile strength was 228 ± 99 MPa, and the modulus of elasticity 3.03 ± 0.26 GPa. The results of tensile strength and modulus of elasticity were consistent with the ranges of previous research data [5,17,28,29], as observed in Table 1.

**Table 1:** Tensile strength and Modulus of elasticity of de fiber coconut.

Authors	Tensile Strength Mpa	Modulus of elasticity Gpa
H. Danso	162	2,49
A. Honda	95–118	8
L. Yan	286–72	2,74-0,34
H. Bui	123,6	

### 3.5 Thermal characterization

The general order of mass loss in natural fibers because of the effect of increasing temperature is: humidity, hemicellulose, cellulose and progressive loss of lignin [6,9].

**Fig. 6:**
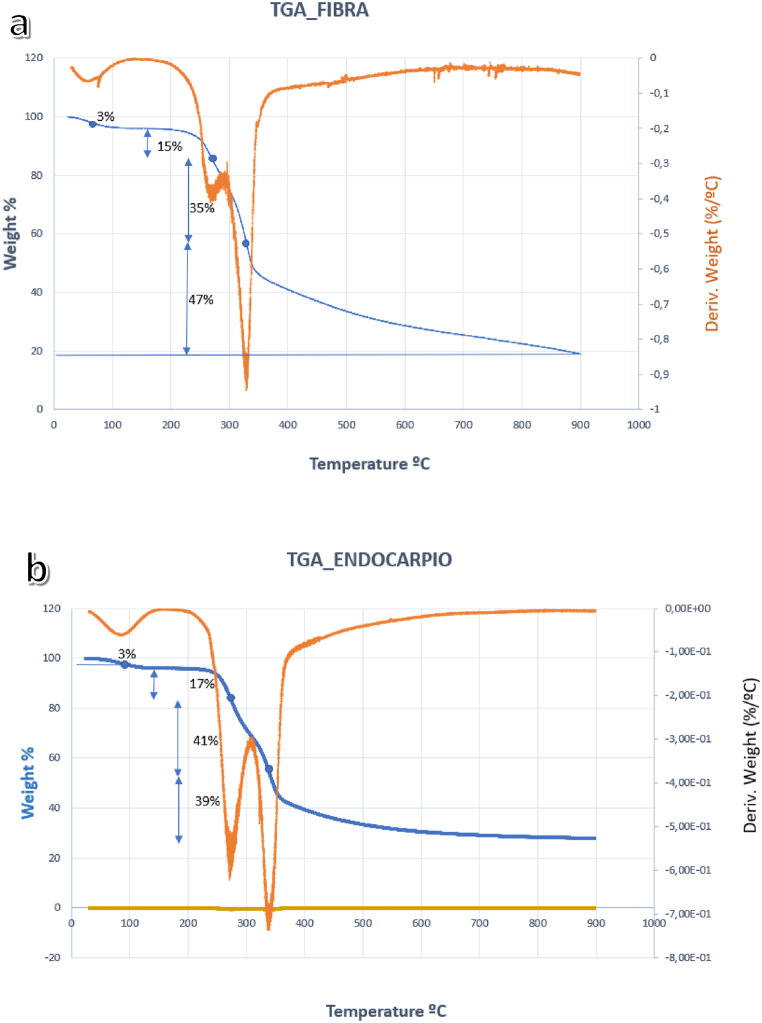
TGA curves, fiber (a), endocarp (b).

The thermogravimetric analysis (TGA) of the fiber shows that at 63 °C there is a mass loss of 3% due to the elimination of humidity; at 120 °C it stabilizes up to 273 °C, where there is a loss of hemicellulose by 15%; and, at 330 °C loss of cellulose by 35%. From this point on the curve, mass losses continue up to 900 °C, Fig. 6a.

Regarding the endocarp, the graph of the derivatives presents peaks of 96 °C, 271 °C and 339 °C, which agree with the TGA curve, since they are inflection points that converge. These points indicate a mass loss because of moisture loss corresponding to 3%, the mass loss related to hemicellulose was 17% and related to cellulose 41%. From 550 °C the loss is continuous, that is, the organic matter, mainly lignin content, ends calcining and the inorganic material remains, until 600 °C when there are no organic losses, Fig. 6b.

The thermogravimetric results were consistent with data from previous investigations [13,[31], [32], [33], [34]].

### 3.6 Chemical characterization

In the chemical composition of the fiber and the endocarp, carbon, oxygen, silicon, potassium and chlorine stand out, as observed in Table 2. The predominant elements in both components of the coconut are carbon and oxygen and there is no clear difference in the amount of each element in the composition of the fiber and the endocarp.

**Table 2:** Chemical composition SEM-EDX, fiber (a), endocarp (b) .

Element	App.Conc.	IntensityCorrn.	Weight%	Weight%Sigma	Atomic%
C K	3.60	0.7643	42.02	2.74	50.95
O K	3.27	0.5861	49.82	2.52	45.35
Si K	0.43	0.9060	4.27	0.45	2.21
Cl K	0.10	0.8105	1.14	0.32	0.47
K K	0.32	1.032	2.74	0.37	1.02
Totals	100				

Element	App.Conc.	IntensityCorrn.	Weight%	Weight%Sigma	Atomic%
C K	4.10	0.8258	41.70	2.60	50.53
O K	3.55	0.5856	50.91	2.43	46.31
Si K	0.27	0.9007	2.49	0.39	1.29
Cl K	0.14	0.8201	1.40	0.32	0.58
K K	0.43	1.036	3.50	0.39	1.30
Totals	100				

Using X-ray fluorescence assay (XRF), coconut fiber samples Fa, Fb, Fc, Fd, Fe, endocarp samples E1, E2, E3, and a sample of strong Portland Holcim cement, Gu type were observed. The results are displayed in Table 3.

**Table 3:** Chemical composition FRX, cement, fiber ash and endocarp.

Componentes	Cement	Fa (500C)	Fb (550C)	Fc (600C)	Fd (650C)	Fe (700C)	E 1 (700C)	E 2 (750C)	E 3 (800C)
	Wt%	Wt%	Wt%	Wt%	Wt%	Wt%	Wt%	Wt%	Wt%
CaO	64.77	4.13	5.57	8.25	5.08	7.58	2.22	2.22	4.31
SiO2	11.06	7.67	9.35	13.88	9.26	13.62	8.61	8.18	12.84
SO3	2.86	1.14	1.07	1.45	0.88	1.40	0.90	0.639	0.951
Al2O3	2.61	0.186	0.34	0.601	0.358	0.452	2.39	2.44	3.75
Fe2O3	1.78	1.07	1.58	0.821	2.29	1.31	11.04	7.31	15.92
MgO	0.891	2.250	4.01	5.62	3.74	5.70	2.11	1.94	2.81
P2O5	0.241	1.96	2.55	3.17	2.10	3.70	2.91	2.24	4.70
K2O	0.143	16.22	24.18	29.04	20.31	35.49	16.58	14.80	31.36
TiO2	0.116	0.016	0.022	0.034	0.019	0.021	0.991	0.418	0.513
SrO	0.083	0.022	0.036	0.046	0.034	0.049	0.017	0.016	0.044
MnO	0.054	0.040	0.055	0.042	0.067	0.053	0.374	0.172	0.308
BaO	0.041	0.022	0.022	0.023	0.021		0.015		0.012
CuO	0.031	0.054	0.047	0.055	0.037	0.045	0.062	0.081	0.222
Cr2O3	0.021	0.256	0.349	0.051	0.603	0.256	0.158	0.295	1.450
ZnO	0.018	0.021	0.033	0.037	0.023	0.043	0.048	0.057	0.129
Cl	0.017	2.300	2.90	2.97	2.88	5.38	1.36	0.488	0.156
PuO2	0.015								
V2O5	0.014			0.002			0.019	0.013	0.024
NiO	0.011	0.019	0.039	0.018	0.041	0.025	0.028	0.040	0.190
Sc2O3	0.003					0.002			
ZrO2	0.002		0.001	0.002			0.013	0.007	0.010
Na2O		2.17	1.57	3.04	2.69	2.46	6.75	4.38	9.26
SnO2		0.004	0.009	0.008	0.007	0.006	0.006	0.004	
MoO3		0.003	0.006	0.003	0.005	0.005	0.003	0.003	0.010
PbO		0.002			0.053		0.002	0.061	0.007
Br		0.025	0.053	0.059	0.045	0.069	0.008	0.002	0.001
WO3			0.014				0.017	0.013	0.032
As2O3					0.007				0.00
I									0.01

The main oxides found in the samples were CaO, SiO_2_, SO_3_, Al_2_O_3_, Fe_2_O_3_, MgO, P_2_O_5_, K_2_O. The percentage of each component present on each sample were compared against the chemical composition of the cement [[30], [35], [36], [37]]. Taking cement oxides as a reference, we observed that the ash with the highest content of these oxides were: in the Fc fiber calcined at 600 °C and Fd at 650 °C, in the E3 endocarp calcined at 800 °C.

## 4 Conclusions

In the cities of Portoviejo and Manta, there is a degree of uncertainty about the handling of coconut residues, which are often incinerated in the open. This circumstance underscores the pressing need to introduce these residues into a circular production system.

In the characterization process, it was found that the coconut fiber exhibits a scaly surface and tubular inner composition, while the coconut endocarp has a smooth surface and dense inner structure. Understanding the relief, shapes, and sizes of these elements is essential to comprehend their adhesion characteristics within the composite matrix.

The physical and mechanical analysis revealed that the coconut fiber possesses significant tensile strength, making it suitable for inclusion in composite material mixtures that need increased resistance. Additionally, the lower density of the fiber and endocarp may help decrease the overall weight of the composite when they are integrated.

Through thermogravimetric analysis, it was observed that the fiber experiences a major mass loss of 35% at 330 °C, and the endocarp undergoes a mass loss of 41% at 339 °C. In both instances, the primary contributor to mass loss was the degradation of cellulose.

The calcination process identified the presence of several oxides, including CaO, SiO2, SO3, Al2O3, Fe2O3, MgO, and P2O5, which are known to be constituents of cement. Research confirms the potential use of coconut husk as a source of silica, and the contribution of SiO2, Al2O3, and Fe2O3 content to the compressive strength of mortar. Further study is needed, however, to determine if coconut ash can be used as a pozzolanic ash.

Coconut fiber and endocarp residues could potentially serve as raw materials in the development of new composite matrices, particularly within the construction sector. Their physical, mechanical, morphological, and thermal properties make them ideal candidates. These materials can enhance load resistance and lower density, contributing to environmental sustainability through the recycling of waste.

## Declarations

### Author contribution statement

Gina Isabel San Andrés: Conceived and designed the experiments; Performed the experiments; Analyzed and interpreted the data; Contributed reagents, materials, analysis tools or

data; Wrote the paper.

Sara Aguilar Sierra: Conceived and designed the experiments; Performed the experiments; Analyzed and interpreted the data.

Graziella Bernardo: Analyzed and interpreted the data; Wrote the paper.

### 4.1 Data availability statement

Data included in article/supp. material/referenced in article.

## Funding Statement

Universidad San Gregorio de Portoviejo Grant: USGP-CU-203-12-2020.

## Declaration of competing interest

The authors declare that the study carried out is specifically a contribution to the investigation without any personal or institutional interest.
