# Supplementary material for: Morphological, physical, and chemical characterization of coconut residues in Ecuador
Source: Heliyon. 2023 Aug 18;9(9):e19267. doi: 10.1016/j.heliyon.2023.e19267 (PMC10477486; doi:10.1016/j.heliyon.2023.e19267)
Supplement: Multimedia component 1 [file mmc1.docx]

Cuestionario

1. Cuántas clases de palmas de coco conoce en el medio

2. Cuál es la diferencia de cada una de estas palmas

3. Qué características tiene el fruto de la palma Manilón

4. Qué características tiene el fruto de la palma Híbrido

5. Qué características tiene el fruto de la palma Criollo

6. Cuál es el fruto de estas palmas que más se vende

7. Cuál es el fruto de estas palmas que menos se vende

Questionary

1. What types of coconut palms are there in this context, as far as you know?
2. What is the difference between each of these palms?
3. What are the characteristics of the fruit of the Manilón palm?
4. What are the characteristics of the fruit of the Hybrid palm?
5. What are the characteristics of the fruit of the Criollo palm?
6. Which fruit from these palms sells the most?
7. Which fruit from these palms sells the least?
